# Novel role of focused airway ultrasound in early airway assessment of suspected laryngeal trauma

**DOI:** 10.1186/s13089-020-00186-3

**Published:** 2020-08-12

**Authors:** Osman Adi, Kok Meng Sum, Azma Haryaty Ahmad, Mahathar Abd. Wahab, Luca Neri, Nova Panebianco

**Affiliations:** 1 Department of Emergency and Trauma, Raja Permaisuri Bainun Hospital, Jalan Raja Ashman (Jalan Hospital), Jalan Raja Ashman, 30400 Ipoh, Perak Malaysia; 2 Department of Anesthesiology & Intensive Care, Beacon Hospital, No. 1, Jalan 215, Off Jalan Templer, Section 51, 46050 Petaling Jaya, Selangor Malaysia; 3 Department of Emergency and Trauma, Kuala Lumpur Hospital, Jalan Pahang, 50586 Kuala Lumpur, Wilayah Persekutuan Kuala Lumpur Malaysia; 4 A.O Niguarda Ca’ Granda’ Hospital, Piazza dell’Ospedale Maggiore, 3, 20162 Milan, MI Italy; 5 Division of Emergency Ultrasound, Department of Emergency Medicine, Hospital of the University of Pennsylvania, 3400 Spruce St, Philadelphia, PA 19104 USA

**Keywords:** Ultrasound, Airway management, Point-of-care ultrasound, Focused airway ultrasound, Laryngeal trauma

## Abstract

**Background:**

Upper airway injury secondary to blunt neck trauma can lead to upper airway obstruction and potentially cause a life-threatening condition. The most important aspect in the care of laryngeal trauma is to establish a secure airway. Focused airway ultrasound enables recognition of important upper airway structures, offers early opportunity to identify life-threatening upper airway injury, and allows assessment of the extent of injury. This information that can be obtained rapidly at the bedside has the potential to facilitate rapid intervention.

**Case presentation:**

We report a case series that illustrate the diagnostic value of focused airway ultrasound in the diagnosis of laryngeal trauma in patients presenting with blunt neck injury.

**Conclusion:**

Early recognition, appropriate triaging, accurate airway evaluation, and prompt management of such injuries are essential. In this case series, we introduce the potential role of focused airway ultrasound in suspected laryngeal trauma, and the correlation of these exam findings with that of computed tomography (CT) scanning, based on the Schaefer classification of laryngeal injury.

## Background

Laryngeal injuries are often undiagnosed in the initial evaluation of the trauma patient. They are rare, with an estimated incidence of one in every 30,000 emergency department admissions [1]. Delayed recognition and intervention may prove fatal in the presence of upper airway obstruction [2].

Blunt laryngeal trauma may present with varying degrees of severity, from mild to life-threatening extremes. A tracheostomy may be required to gain airway access distal to the site of injury. A systematic classification and management approach of blunt laryngeal trauma is crucial to guide early decision-making and improve patient outcome in the emergency department.

Current standard of care for laryngeal trauma is determined according to the Schaefer classification of laryngeal injury. Schaefer group 1 and group 2 with minor endolaryngeal injuries can be managed conservatively with observation, antibiotics, steroids, voice rest and anti-reflux medications. However, for more severe Schaefer type 3–5 injuries, open surgical repair will be required to secure a definitive airway [1, 2].

Good history taking, detailed clinical examination and a high index of suspicion are critical in the diagnosis of laryngeal trauma. The diagnosis can be aided using flexible nasendoscopy by direct visualization of the airway. CT scanning of the neck is still considered the gold standard to grade the severity of the injury and to direct appropriate management. Obtaining a timely CT scan may often be challenging due to logistical problems, primarily availability of radiology service support especially in resource-limited area, and stability for patient transfer.

Of late, studies have integrated the use of upper airway ultrasound into point-of-care ultrasound examination, a paradigm shift in upper airway assessment [3–5]. The incorporation of ultrasound into the diagnostic arm may expedite the intervention process by removing some logistics problem and provide rapid information to guide timely management.

We discuss the role of focused airway ultrasound in upper airway trauma performed by point-of-care ultrasound trained emergency physician and propose a focused airway ultrasound classification in relation to Schaefer classification of laryngeal injury.

## Case presentation

### Case 1—endolaryngeal hematoma without detectable fracture (Schaefer group 1)

**Fig. 1:**
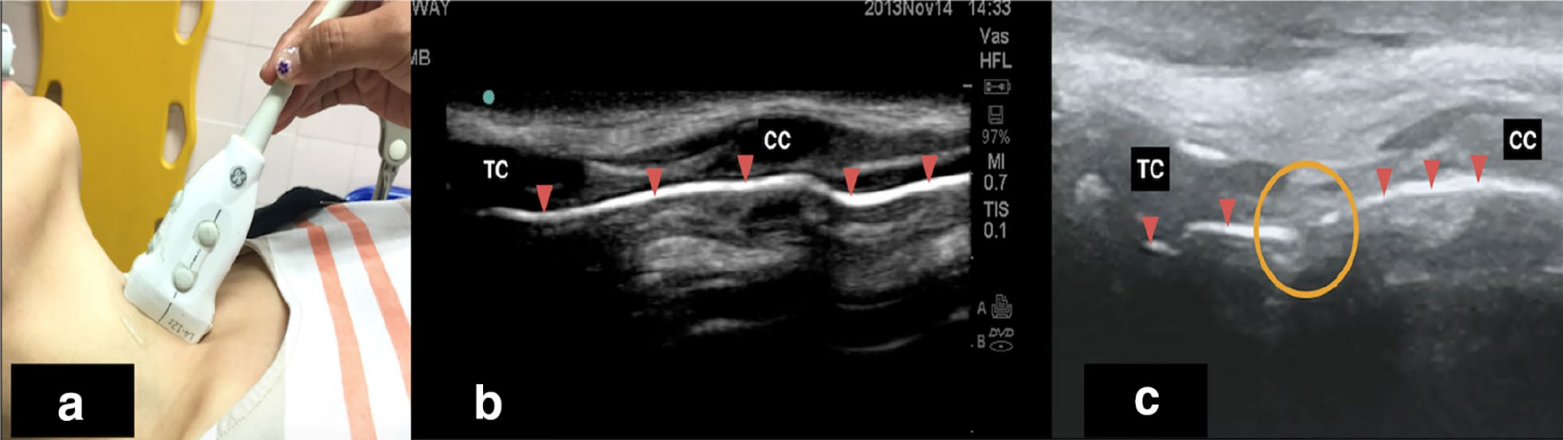
**a** Surface landmark of probe position (in longitudinal view) in relation to scanning the area for **b** and **c**. **b** Normal sonoanatomy of upper airway showing the relationship between thyroid cartilage, cricoid cartilage, air–mucosal interface and surrounding soft tissues. A continuous and undisrupted line of air–mucosal interface seen as hyperechoic line (arrowhead) **c** Airway ultrasound showed disruption air–mucosal interface without obvious detectable laryngeal fracture (circle). SM: sternocleidomastoid muscle; TC: thyroid cartilage; CC: cricoid cartilage

A 24-year-old male presented with neck swelling without signs of respiratory distress after a traumatic blunt neck injury. There was swelling of the anterior neck without palpable crepitus. Airway ultrasound showed disruption of the air–mucosal interface suggesting endolaryngeal disruption (Fig. 1b). CT scan confirmed the diagnosis of endolaryngeal disruption without cartilaginous fracture. The patient was conservatively managed and discharged well on the third day.

### Case 2—undisplaced thyroid cartilage fracture (Schaefer group 2)

A 66-year-old motorcyclist, was injured in a collision with a van. He presented with mild neck pain, difficulty in breathing, hoarseness, dysphagia and odynophagia. He had stridor, and his neck was swollen and tender with subcutaneous emphysema.

**Fig. 2:**
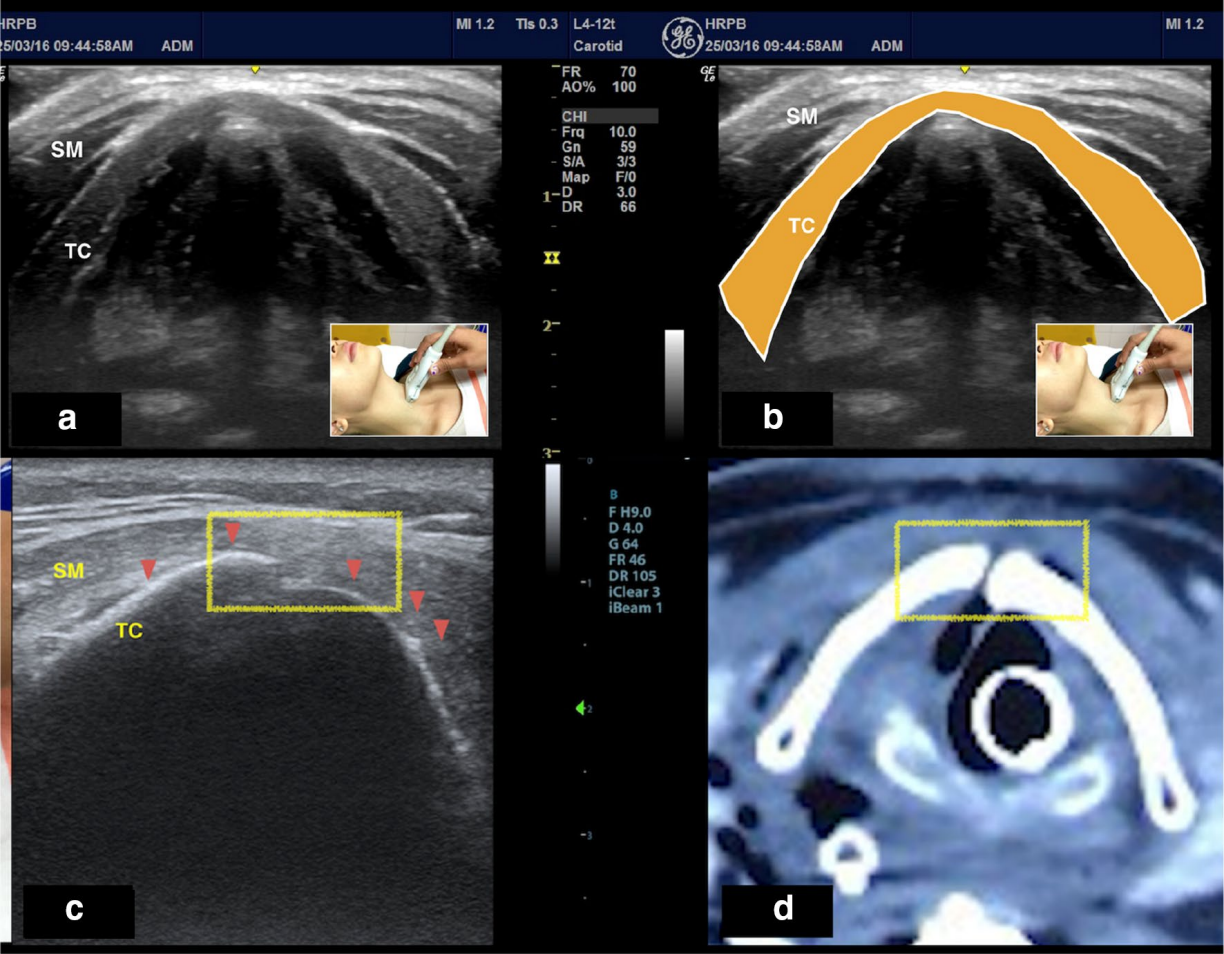
**a**, **b** Surface landmark of the probe position and ultrasound image of the normal thyroid cartilage (in transverse view). **c** Airway ultrasound showing undisplaced fracture of the thyroid cartilage (box) and disruption of the anterior cortex of the thyroid cartilage (arrowhead**). d** A Computerized tomography (CT) scan image shown right thyroid lamina fracture with surrounding prevertebral edema and hematoma. SM: sternocleidomastoid muscle; TC: thyroid cartilage

Airway ultrasound using a 15-MHz linear transducer found discontinuity of the anterior cortex of thyroid cartilage with minimal surrounding tissue edema, consistent with Schaefer group 2 (Fig. 2c). CT scan confirmed the ultrasound findings, showing a defect in the posterolateral wall of the trachea and the esophagus, with fracture of the right anterior lamina of the thyroid cartilage and superior cornu of the left thyroid cartilage (Fig. 2d).

He was immediately intubated and was started on intravenous dexamethasone to reduce inflammation and edema, a proton pump inhibitor to prevent reflux and laryngeal irritation, nebulized adrenaline and a prophylactic antibiotic in the emergency department. The patient was managed conservatively and was discharged well from intensive care unit on the fifth day post trauma.

### Case 3—displaced thyroid cartilage fracture (Schaefer group 3)

A 28-year-old male martial art athlete was kicked by his opponent and sustained a blow to the anterior part of the neck. He complained of pain, dysphagia and hoarseness. There was an abrasion to the anterior part of his neck, which was tender to palpation with localized crepitus.

**Fig. 3:**
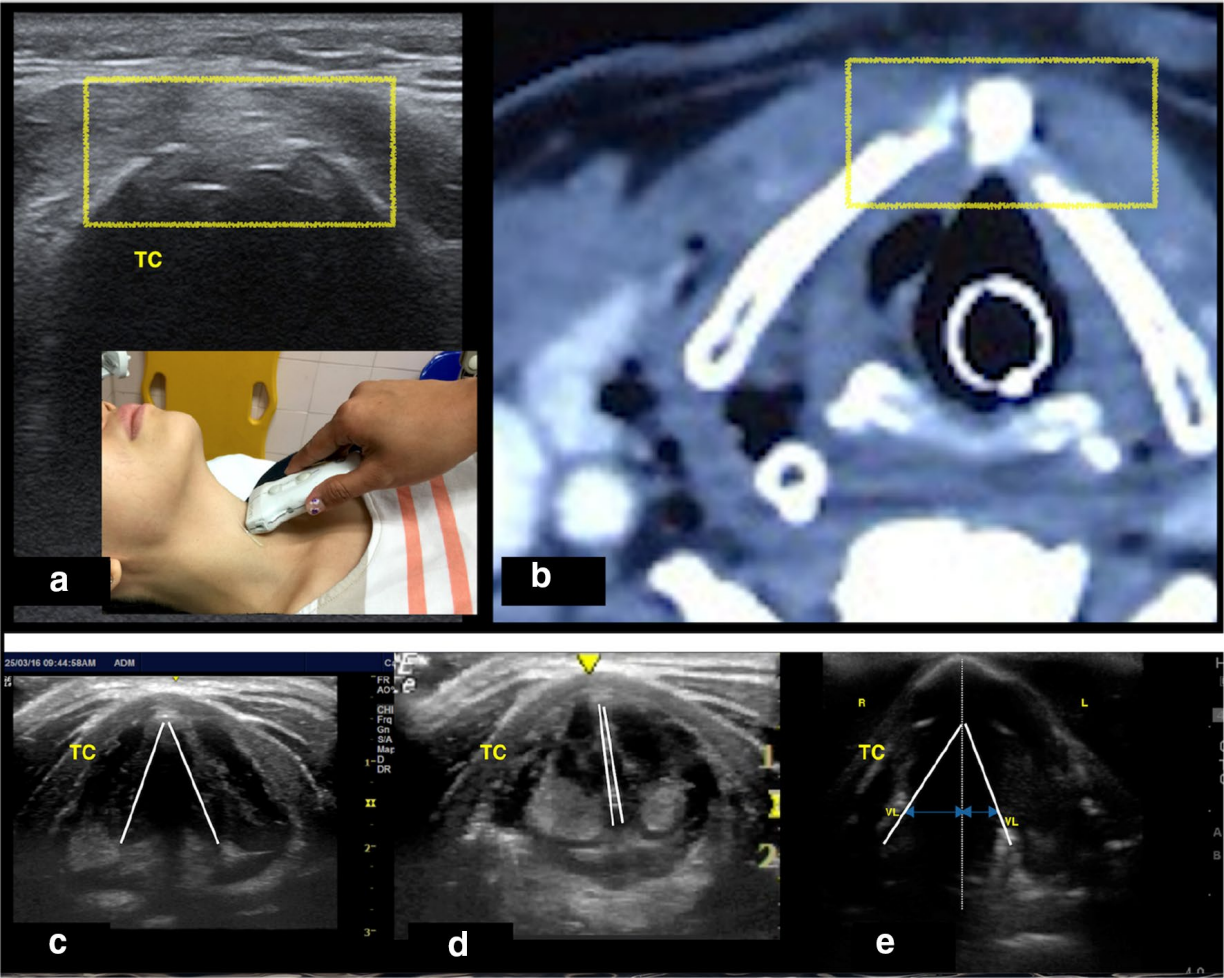
**a** Image of focused airway of displaced thyroid cartilage fracture and disruption of anterior cortex of the thyroid cartilage (box). **b** A computerized tomography (CT) scan image showing defect in posterolateral wall of trachea with fracture of right anterior lamina of thyroid cartilage and superior cornu of left thyroid cartilage. **c**, **d** Assessment of vocal cord mobility can be done by looking at the movement of vocal ligament (white line) during abduction and adduction. **e** Blue arrow indicates reduced movement of the right vocal ligament (white line) to the midline during adduction compare to the left vocal ligament. R: right; L: left; VL: vocal ligament, TC: thyroid cartilage

Bedside airway ultrasound revealed a displaced fracture of the thyroid cartilage, disruption of anterior cortex of thyroid cartilage with surrounding mixed echogenicity denoting endolaryngeal edema (Fig. 3a and Additional file 1: Video S1) and paralyzed right vocal cord (Fig. 3e), consistent with Schaefer group 3.

Direct visualization using a flexible fibreoptic scope revealed an edematous and medially deviated right arytenoid with paralyzed and erythematous right vocal cord. He was intubated and intravenous dexamethasone, proton pump inhibitor, nebulized adrenaline and prophylactic antibiotic were initiated early in the emergency department. The patient was sent for CT neck after stabilization, which showed a defect in the posterolateral wall of the trachea with a displaced fracture of right anterior lamina of thyroid cartilage and superior cornu of left thyroid cartilage, consistent with Schaefer group 3 (Fig. 3b), and that found on bedside ultrasound. The patient was stable throughout his entire hospitalization after open surgical repair and was allowed home on day nine with outpatient follow up.

### Case 4—displaced thyroid cartilage fracture (Schaefer group 3)

A 35-year-old male lorry driver hit his neck against the steering wheel when he thrown forwards during a head-on collision. He presented with neck pain, severe swelling over the whole anterior region of the neck, stridor, hypoxia and a compromised airway. The patient was immediately intubated and ventilated.

**Fig. 4:**
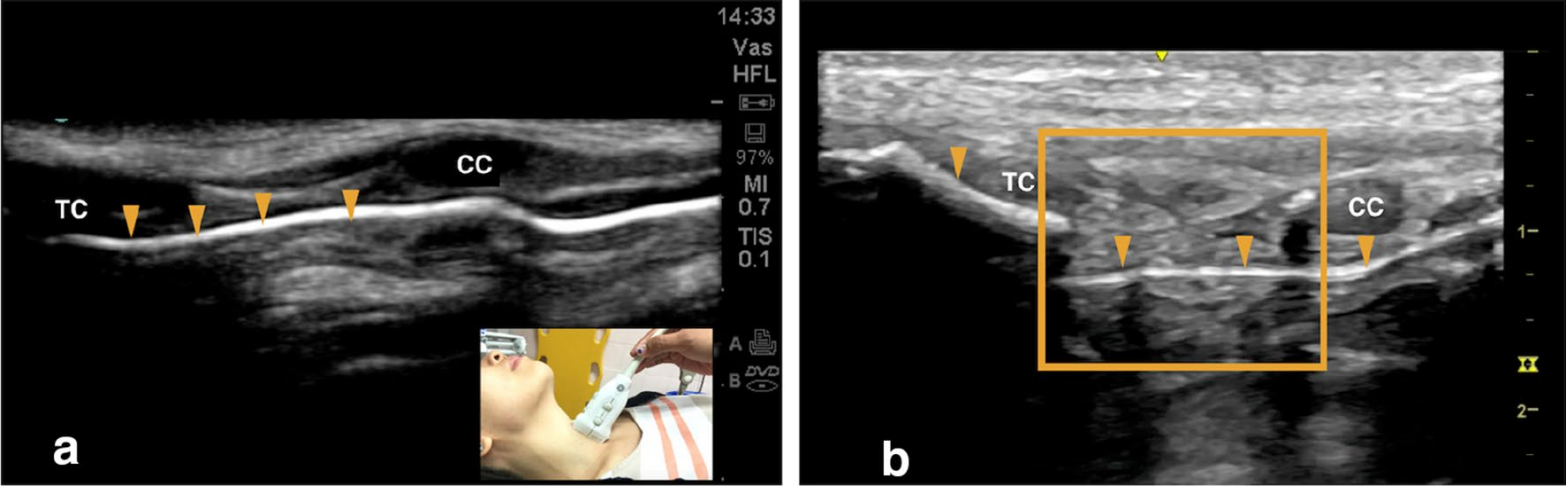
**a** Normal sonoanatomy in longitudinal scan showing continuous and intact air–mucosal interface (arrowhead) in relation to thyroid and cricoid cartilage. **b** Airway ultrasound image in longitudinal scan showing disruption of the air–mucosal interface (arrowhead) and formation of endolaryngeal hematoma and fracture cricoid cartilage (box). TC: thyroid cartilage; CC: cricoid cartilage

Focused airway ultrasound showed disruption of the air–mucosal interface, a displaced thyroid cartilage fracture with formation of endolaryngeal hematoma and a cricoid cartilage fracture (Fig. 4b). He was treated as Schaefer group 3 and was started on intravenous dexamethasone, proton pump inhibitor, nebulized adrenaline, prophylactic antibiotic and open surgical repair was planned.

CT scan revealed a defect in the posterolateral wall of the trachea with a displaced fracture of the left anterior lamina of thyroid cartilage and hematoma surrounding the thyroid and cricoid cartilages. This confirmed the classification and injury details categorized under Schaefer group 3, which correlated with that of ultrasound assessment. Hospital stay was uneventful, and he went home after 2 weeks, to be reviewed in the outpatient department.

## Discussion

The ABCs of trauma, a mantra that prioritizes the primary survey starts, with “A” for airway. In this case series, we describe four encounters in which ultrasound of the upper airway was performed for suspected laryngeal trauma and correlated with CT scan assessment of severity based on the Schaefer classification of laryngeal injury.

Upper airway obstruction as a consequence of laryngeal injury may be catastrophic. Apart from blunt force trauma, iatrogenic injuries can occur after percutaneous dilatational tracheostomy, fiberoptic bronchoscopy, airway manipulations and procedures, even tracheal intubation [6, 7, 8]. The lack of correlation between symptoms, physical findings and severity of laryngeal injury may result in delayed recognition of such injuries. Additionally, patients with laryngeal injury are at risk of false passages, transforming an incomplete fracture to total separation of the upper airway, converting mild upper airway obstruction to complete obstruction especially in undiagnosed laryngeal trauma [9–11]. For these reasons upper airway ultrasound may play an important role in the early assessment for laryngeal injury.

For the past three decades the internationally accepted Schaefer classification of laryngeal injury stratification system has been used to categorize laryngeal injury. This classification not only categorizes, but it also guides management. It divides the management plan into 2 categories; non-invasive or conservative airway management for group 1 and group 2 injuries, and invasive airway management for higher grade injuries (group 3 to group 5). Further study is needed to determine if focused upper airway ultrasound can reliably be used to determine injury grade [1].

While the authors could not find previous publication of ultrasound assessment for upper airway injury in trauma, prior research by Osman et al. and You-Ten et al. briefly illustrated the usage of airway ultrasound in a step-by step manner to delineate the normal sonoanatomy of the upper airway such as thyroid cartilage, epiglottis, cricoid cartilage, cricothyroid membrane, tracheal cartilages, esophagus and the surrounding soft tissues [3–5]. Schick et al. published promising evidence on the use of airway ultrasound in the emergency setting to identify airway edema and impending threats to the airway [12]. Airway ultrasound can also be used to assess laryngeal edema in the post-extubation period. [13–15]. Kameda et al. [16] identified airway edema as hypoechoic thickening of the tracheal wall on airway ultrasound in a patient with inhalational burns. The findings on the sonogram were later confirmed by CT scan, demonstrating good correlation between focused ultrasound and CT Scan.

Cheng et al. [17] found good correlation between sonographic visualization of abnormal vocal cords movement and laryngoscopic examination. They demonstrated that clinician-performed airway ultrasound is an accurate screening tool for preoperative assessment of vocal cord movement.

Upper airway ultrasound findings that correlate with the Schaefer Classification System may be especially relevant in hemodynamically unstable patients where CT imaging is not feasible. While larger trials are needed, we propose that focused airway ultrasound can be used to correlate with the Schaefer Classification System (Table 1) and therefore propose it be assimilated into the work-up of laryngeal trauma.

**Table 1:** Proposed focused airway ultrasound findings in correlation to the Schaefer Classification System and standard management of laryngeal injury

Group -Based on Schaefer classification [1]	CT scan findings -Based on Schaefer classification [1]	Focused airway ultrasound findings	Standard management and intervention [1, 2]
Group 1	Minor endolaryngeal hematoma or laceration without detectable fracture	Endolaryngeal hematoma without detectable fracture	Supportive care including observation, antibiotics, humidified air, supplemental oxygen, anti-reflux medications, voice rest and early steroid administration. *Patients with Group 2 injuries should be serially examined, since the injuries may worsen or progress with time. Occasionally group 2 injuries may require a tracheotomy
Group 2	Edema, hematoma, minor mucosal disruption without exposed cartilage, nondisplaced fracture noted on CT	Edema, endolaryngeal hematoma, minor mucosal disruption without exposed cartilage, nondisplaced fracture Mucosal hematoma/edema Nondisplaced fracture of cartilage framework	
Group 3	Massive edema, mucosal tear, exposed cartilage, cord immobility, displaced fracture	Edema, cord immobility, displaced fracture Vocal fold immobility Obvious displaced fracture	Direct laryngoscopy, esophagoscopy and immediate open surgical repair is deemed necessary due to extension of injuries
Group 4	Addition of more than two fracture lines or massive trauma to laryngeal mucosa	Addition of more than two fracture lines Comminuted fracture of laryngeal cartilage framework	
Group 5	Complete laryngeal separation		

Possible advantages of focused upper airway ultrasound in the diagnostic classification of blunt laryngeal trauma are:

- In centers without CT scan capabilities—focused airway ultrasound can complement emergency department triage protocol to enable early airway management planning in blunt laryngeal injury.
- In centers with CT scan facility—focused airway ultrasound can hasten airway management planning prior to airway catastrophe during an emergency situation when transfer to the radiology suite is deemed unsuitable.
- In resource-limited situation such as—scarce resources, remote area, and humanitarian medical mission in environmental disasters and war-torn regions—focused airway ultrasound can supplement disaster and transfer protocol facilitating decision for early airway intervention while preparing for emergency transfer.
- It has the added advantage of real-time visualization of dynamic vocal cords function.

### Limitations

Ultrasound evaluation of the airway may not be practical in every case. Subcutaneous emphysema, posterior laryngeal injury, cartilage calcification and foreign bodies may result in artifacts interfering with ultrasound images and interpretation. Furthermore, ultrasound techniques and interpretation are operator-dependent, and have a steep learning curve. Adequate competency training and reproducibility is important to standardize findings.

### Future directions

Further studies to identify optimal management strategies for patients with laryngeal injury are required. Areas of interest include:

- Validation studies comparing accuracy of focused airway ultrasound to CT scan findings across a range of injury types.
- Management outcome, cost effectiveness, accuracy and time of diagnosis of focused airway ultrasound compared to existing radiological modalities.
- Reproducibility of results by different operators at different stages of seniority and proficiency level, their abilities to accurately detect pathology and studies on learning curve of this procedure.

## Conclusion

Ultrasound assessment of the upper airway is a promising adjunct in the rapid evaluation of patients with suspected laryngeal trauma. Early diagnosis and injury classification stratification with point-of-care ultrasound may play an important role in trauma patient care, particularly those too unstable for CT imaging or when advanced imaging is unavailable.

## Supplementary information

**Additional file 1: Video S1.** Bedside airway ultrasound revealed a displaced fracture of the thyroid cartilage, disruption of anterior cortex of thyroid cartilage with surrounding mixed echogenicity denoting endolaryngeal edema.

## Data Availability

The material during the current case series is available from the corresponding author on reasonable request

## Abbreviations

POCUS: Point-of-care ultrasound
CT: Computed tomography
Sm: Sternocleidomastoid muscle
Tc: Thyroid cartilage
Vm: Vocalis muscle
VL: Vocalis ligament
AC: Arytenoid cartilage
